# Home Colonisation

**Published:** 1888-04-28

**Authors:** 


### Home Colonisation.

Of all the schemes which have been suggested for the amelioration of the condition of the unemployed poor, there is none that has a more fascinating sound than that of Home Colonisation. At the same time none has been so scornfully satirised by the people who pooh-pooh everything, and who, comfortably seated in their easy chairs, settle all social problems by remarking that the Scriptures say, "The poor ye have always with you," as if this quotation from the Gospel contained indeed some esoteric good tidings for the poor. In spite of these good folks, however, the attempt to raise the poor at least above the level of starvation and beggary will continue to be made by all who recognise their social position as members of the great brotherhood of humanity. Many plans have been and will be tried; not all of these will attain the end they seek; and those who have promoted them will have to seek consolation in remembering "how far high failure overleaps the bound of low successes."

Allusions to "high failures" in similar fields are among the most popular arguments against the present scheme of Home Colonisation, brought forward by Mr. Herbert Mills, and already spoken of in our columns. But the experience of the past forms the wisdom of the future; and that organisations similar, but not the same, have failed, is no reason for assuming that this, formed under more favourable conditions, is doomed also. *Fiat experimentum, sat est,* is not the motto of progress.

Let us state the argument for Home Colonisation. We have among us, as we all know, a large number of unemployed men and women, who, by reason of poverty, are liable to drift into either the pauper or the dangerous classes, to the manifest loss and possible danger of the State. Some of these would rather beg or sin than work, and the Home Colonisation Society does not propose to deal with these. But the case of those who, willing to work and competent to fulfil some branch of useful labour, are, for lack of employment, liable to be forced to beg or steal, is different, and it is worth while to do something to save them from coming to such a pass. The specific usually recommended is emigration; and a very good remedy it is, but it has one great disadvantage, which is now being severely felt in the districts where emigration has been most encouraged—namely, that the strong go away, and the weak are left behind. Emigration means, in the long run, exporting men, and often it is the flower of our manhood—those who can "make their way" in face of difficulties—who go, while there are left in the native land the weak and incompetent, as well as a larger proportion of women than nature meant.

Meanwhile, there are in England hundreds of acres of land going out of cultivation, because the farmers cannot work them at a sufficient profit. Nothing, as Mr. Mills pointed out at a recent meeting, tends more to the ruin of an empire than this neglect of the land. With Rome, Babylon, and Egypt, when agriculture was neglected as unprofitable, it was the sign of degeneracy, of effeminate luxury in the rich, and that sullen, hopeless poverty in the poor that makes for revolution. Ruskin rightly says that the true wealth of a nation lies not in its coal or iron, but in its men; and a country where there are no tillers of the soil, or where these are growing fewer, is not increasing its wealth of manhood.

But why are these acres lying fallow, falling like a burden on their landlords' hands, till they in turn plead poverty? Because the soil is poor and barren? No; the wheat-crop of England, acre for acre, still exceeds that of America. The reason land does not pay is because of the difficulty of finding a market for its produce, and because those who have farmed it wished to gain more from it than the necessaries of life. The land of our country will still provide these, and in the scheme of the Home Colonisation Society there is no attempt to make it do much more. It is not a plan for the aggrandisement of any one individual; it professes to do nothing more than give a sufficiency of food and clothing to people who are, or are likely presently to become, a burden or a danger to society.

The scheme, in brief, is this: The Home Colonisation Society will purchase an estate containing 500 acres at a price not exceeding £10 an acre. This tract of land will be chosen rather on account of its fertility than because it is near a railway, a question which has hitherto been very important in the choice of farms, but will now be inoperative, because the colonists themselves will consume the greater part of what the colony produces. Necessary tools and agricultural stock must then be purchased, and matrons and foremen of departments engaged. The first work must of course consist of the building of houses, which would include, not only cottages, but a central building containing kitchen, dining-room, reading-room, recreation-room, and lecture-hall; for the colony is not meant to be a mere chance collection of isolated individuals, but a community in the real sense of that much-abused word. Most of the cooking, the three meals a day which Mr. Mills considers necessary for every man and woman, will be cooked in the general kitchen and served in the public dining-room; but the women of the society will be allowed, if they prefer it, to prepare the evening meal in their own home

The first company of colonists should consist of builders, carpenters, agricultural labourers, gardeners, bakers, cooks, and laundresses; soon afterwards tailors, shoemakers, dressmakers, and needlewomen, and finally spinners and weavers would be added. This list of workers should be studied by those who oppose the scheme, and who seem to assume somewhat rashly either that 500 agricultural labourers are to be set to do all manner of unaccustomed work, or that a collection of waifs and strays, able-bodied and the reverse, are to be set to do their untrained best or worst at farming. Neither hypothesis is a true or even moderately just one. The colony will be modelled on those communities which formed the first settlements in New England. Each band that set out had its baker, tailor, and shoemaker, its representative of all those handicrafts which are essential to the welfare of a community, as well as agriculturists. Each of these works for all the others, giving the result of one kind of labour and receiving that of another; in this way the colony will be self-contained, not being obliged to go outside its own bounds to obtain the necessaries of life. No worker will need to compete in the open market for the sale of his produce in order to gain money which he will expend in buying the produce of his neighbours. The interchange of work will be direct, and the profit of the go-between, which now causes the large difference between the price paid to the worker and the price paid by the purchaser, would be done away with.

The law, inevitable in a civilisation like ours, of the division of labour, will be strictly adhered to. Each man and woman will do just what he or she has been trained to do; but the difference will lie in their not having to go far afield seeking for a market—to find which is often a more difficult task than to produce the work. This is evidenced by the fact that most manufacturing firms pay their travellers more—sometimes three or four times as much—as they do their workers and managers. When the market is secured, the labour problem is more than half solved.

The intention is that all clothing should be made by the women of the colony, to whom also would be assigned all cooking and fruit-preserving, all washing and house-cleaning, and most of the clerical work. There would thus be afforded a loophole of escape from starvation for those women who now crowd the labour market in such numbers that their mere presence lowers the rate of wages to a point which makes those who have work but little better off than those who have none. How important this is for the welfare, not merely of the women themselves, but of the whole community, it is almost impossible to say. Starving women are a danger to the State as real, if not as obvious, as starving men; and on this point we cannot do better than quote Mr. Mill's words: "Although they do not meet in organised bodies and menace the peace of our cities as do the men, yet they suffer more keenly than men from low wages, lack of food, and lack of work; and they endure temptations when they are half-fed and ill-housed, the bitterness of which men seldom attempt to realise."

The colonists are to give five hours' work daily to the society, for which they are to receive no payment in money, but instead, a rent-free house, three good meals daily, a suit of clothes every year, education for their children—100 children are to be among the first colonists, the remainder consisting of married couples and unmarried able-bodied men and women in reasonable proportions—an allotment of land, consisting at first of one-third of an acre, and lessons in the arts of bee-keeping, mushroom culture, basket and mat making, and handicrafts like these.

Why is this done? Honey and mushrooms cannot be included among those necessaries of life which are all the Society promises its colonists. No; but it does not expect them to be content with mere necessaries: it will not try to cultivate in them that contentment which is fatal to progress. And as five hours' work a-day is less than an able-bodied man or woman can accomplish, the colonists will be encouraged to undertake in their off-time such additional work as will bring them in money to buy the little luxuries of life. In this way individual industry and energy will tell. Those who do the most extra work will be the best off in this community, as in all others; but none who are willing to do their five hours' work daily need fear hunger or cold. This is all the Society undertakes to do; but it will encourage all individual efforts to gain more than this, and especially to have the desired advantages provided as well as consumed by the colonists. Thus the young women may set up shops for the sale of tobacco, newspapers, and the like; the men would do extra work of the kind allotted to them, and sell the produce of it in one fashion or another. Thus the wheelwright might make a cart in his off-time, in which he could convey the farm or dairy produce of some of his neighbours to market, receiving from each a small sum as carrier's fee. Such subsidiary branches of industry would arise in time of their own accord, and keep the community from drifting into that state which is commonly, though inaccurately, described as socialism, in which skilful and industrious work for the benefit of the incompetent and the lazy.

And now, how much would this scheme cost if carried into practice? A large sum—£25,000. But those who shake their heads at this should remember that if these five hundred people, men, women, and children, were in the workhouse—whither, or else to the prison, they are inevitably drifting if they remain unemployed—they would cost the ratepayers £25,000 in two years. It will cost no more to put them on the high road to independence and comfort, than to keep them for two years in a condition which by general consent is looked upon as wretched and degrading, which loosens the bonds of family life, and develops an unintellectual and dependent habit of mind, which is often hereditary. It is worth risking something to keep our, as yet, self-respecting poor from degenerating into paupers. And if this money were subscribed it would be expended for the distinct and continuous profit of the poor, with the least possible waste, whereas our present system of poor-law relief seems often to strive for the other extreme. In the West Derby Union Mr. Mills found that of £100,000 raised by the poor-rates, £49,000 was expended on the maintenance of the poor, and £51,000 in salaries of various kinds. The Home Colonisation Committee purposes to act with considerably more economy than this.

One thing is wanted besides money, one thing is equally essential with it—a man who will manage the colony in no hireling spirit, but give himself heart and soul to its welfare; a man whom all will obey because they know his rule to be just and wise. Even long-established republics need a president; young states and small states flourish best under the salutary despotism of a wise dictator. If such a man is forthcoming we do not doubt that Home Colonisation will succeed, and will do much to prevent that expatriation of our goodliest men, which is more a matter for regret than the fact that we have colonies to receive them is a matter for thankfulness.

Ruskin shows himself no vain theoriser, but a true philosopher, and perhaps the wisest political economist of our day, when he says: "There is no wealth but life—life, including all its powers of love, of joy, and of admiration. That country is richest which nourishes the greatest number of noble and happy human beings." For such wealth, and not wealth of coal and iron, nor of stocks and shares, let us strive, if we would have our country the Merrie England of the olden times.

